# Renal Cancer Stem Cells: Characterization and Targeted Therapies

**DOI:** 10.1155/2016/8342625

**Published:** 2016-05-15

**Authors:** Anna Julie Peired, Alessandro Sisti, Paola Romagnani

**Affiliations:** ^1^Excellence Centre for Research, Transfer and High Education for the Development of DE NOVO Therapies (DENOTHE), 50139 Florence, Italy; ^2^Department of Biomedical, Experimental and Clinical Sciences, University of Florence, 50139 Florence, Italy; ^3^Nephrology Unit, Meyer Children's University Hospital, 50141 Florence, Italy

## Abstract

Renal cell carcinoma (RCC) is a major neoplasm with high incidence in western countries. Tumors are heterogeneous and are composed of differentiated cancer cells, stromal cells, and cancer stem cells (CSCs). CSCs possess two main properties: self-renewal and proliferation. Additionally, they can generate new tumors once transplanted into immunodeficient mice. Several approaches have been described to identify them, through the expression of cell markers, functional assays, or a combination of both. As CSCs are involved in the resistance mechanisms to radio- and chemotherapies, several new strategies have been proposed to directly target CSCs in RCC. One approach drives CSCs to differentiate into cancer cells sensitive to conventional treatments, while the other proposes to eradicate them selectively. A series of innovative therapies aiming at eliminating CSCs have been designed to treat other types of cancer and have not been experimented with on RCC yet, but they reveal themselves to be promising. In conclusion, CSCs are an important player in carcinogenesis and represent a valid target for therapy in RCC patients.

## 1. Introduction

Renal cell carcinoma (RCC) constitutes the most common form of renal neoplasms, comprising more than 90% of cases in adults of both sexes, with an occurrence 2 to 3 times higher in men than in women. The incidence increases after 40 years of age, as for all tumors of epithelial origin, and decreases after 75 years in both sexes [1, 2]. RCC is classified into several different subtypes based on the pathological features. The most common subtype is clear cell RCC (ccRCC), followed by papillary RCC (pRCC), chromophobe, and collecting duct RCC. The 2013 Vancouver classification includes a total of 17 morphotypes of renal parenchymal malignancy and two benign tumors [3–6]. RCC is becoming more commonly diagnosed worldwide and, consequently, mortality is decreasing in the most developed settings. However, it remains prevalent in low- and middle-income countries, where access to and the availability of optimal therapies are likely to be limited [2]. Surgical management of the primary tumor remains the gold standard of RCC treatment. Nevertheless, RCC high metastatic index and resistance to radiation and chemotherapies have led to the development of new therapeutic agents that target the tumor vasculature or that attenuate the activation of intracellular oncogenic pathways [7].

**Figure 1:**
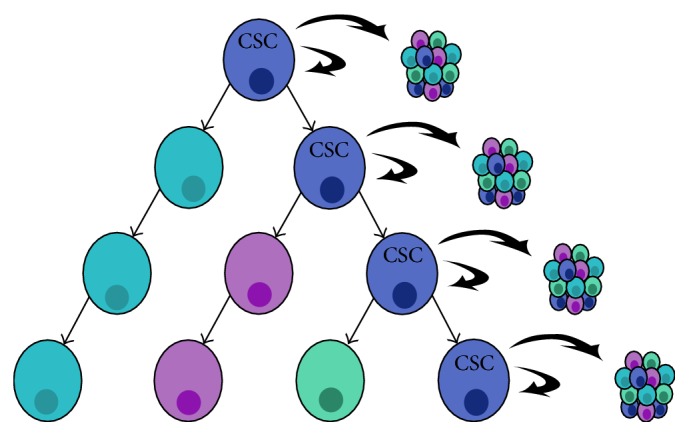
Cancer stem cell model. Tumor cells form a heterogeneous structure and only the cancer stem cells (CSCs) have the ability to self-renew and differentiate into different cell types. CSCs can form new heterogeneous tumors following transplant.

Tumors are heterogeneous structures composed of different types of cancer cells, each cell population presenting variations in metabolism, receptors, and ligands expression and epigenetic chromatin structure alterations [8–13]. Identifying specific cell types within a tumor that either initiate or maintain tumorigenesis provides valuable information and allows a better understanding of tumor biology, as well as the development of novel treatments. The cell of origin of cancer, or tumor-initiating cell (TIC), is a normal cell that sustains mutations leading to tumor formation [14]. The cells that maintain tumor growth and propagation are the cancer stem cells (CSCs) [15]. However, the use of the TIC or CSC terminology is sometimes redundant, as the distinction between the two populations is blurry. CSCs possess two main characteristics: self-renewal and multipotency capacity. Self-renewal allows unlimited cell division and maintenance of the stem cell pool in the tumor. Multipotency permits CSCs to divide and create a progeny that keeps dividing until they yield terminally differentiated, specialized cells [16]. Additionally, CSCs can by themselves originate a tumor mass indefinitely, following transplant into immunodeficient mice (Figure 1). As a matter of fact, the cancer transplantation assay constitutes the gold standard in identifying CSCs as it can provide evidence of both self-renewal and multilineage potency of CSCs [17]. It consists in implanting a putative CSC population into immunodeficient mice, and if the cells give rise to serially transplantable tumors that recapitulate the cellular heterogeneity of the parental tumors, they can conclusively be qualified of CSCs. On the other hand, TICs can be defined by lineage tracing assays, which allow defining the cell of origin of transformation in mouse models [17]. The use of cell-specific promoters allows distinct cell subpopulations to be labeled, allowing tracking of single-cell-derived clones. This assay permits us to assess the fate of individual cells that undergo transformation and form a tumor and to definitively identify them as TICs. Consecutively, labeled TICs can be sorted and used in serial transplantation to evaluate their CSC properties.

Various hypotheses exist to describe the origin of TICs/CSCs, such as accumulation of several mutations during their lifespan or reprogramming of tumor cells through dedifferentiation by hypoxia and/or epithelial-to-mesenchymal transition (EMT) [18–20]. Several mechanisms confer CSCs resistance to radiation and chemotherapeutic treatments, including their quiescent state, their presence in hypoxic microenvironments, upregulation of damage response mechanisms, and their increased drug efflux potential [16, 21]. Conventional therapy does not target the CSC population in RCC, and despite an initial tumor size reduction the patient relapses. A better identification and characterization of CSCs would allow the development of new drugs to selectively eradicate this population in RCC patients.

## 2. Cancer Stem Cells in Renal Cell Carcinoma

**Figure 2:**
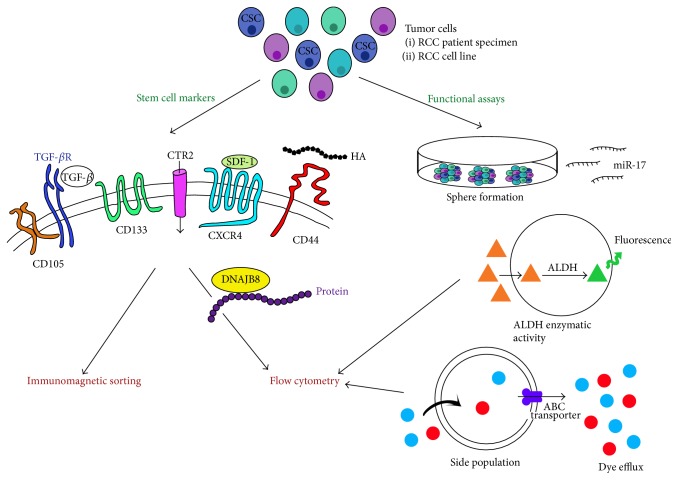
Identification of cancer stem cells using stem cell markers and functional assays. Several techniques have been described to identify cancer stem cells (CSCs) and to isolate them by immunomagnetic sorting, flow cytometry, or cell culture. HA: hyaluronic acid; red circle: Hoechst 33342; blue circle: Rhodamine 123; ALDH: aldehyde dehydrogenase; orange triangle: BODIPY-aminoacetaldehyde (substrate); green triangle: BODIPY-aminoacetate.

Renal tubular cells have been extensively described as the cellular origin of RCC [19]. Numerous studies have attempted to isolate and characterize a population of CSCs among tubular cells, using either stem cell markers or functional assays (Figure 2) [18, 22, 23].

### 2.1. Stem Cell Markers

Several molecular markers have been used to identify CSCs and constitute valuable tools for tumor detection and diagnostic, prognostic, and predictive values, as well as determination of therapeutic targets [24]. Of note, no marker has been found so far that would be expressed only in CSCs [25].

#### 2.1.1. CD105

CD105, also called endoglin, is a surface transmembrane molecule that is part of the transforming growth factor-*β* (TGF-*β*) receptor complex. It regulates cell proliferation, differentiation, and migration and has an important role in angiogenesis [26]. In 2008, Bussolati et al. proposed CD105 as a marker for tumor-initiating stem cells [27]. Cells from patient specimens of RCC were labeled with CD105 and separated by magnetic sorting. They represented less than 10% of the total tumor cells and expressed the mesenchymal stem cell markers CD44, CD90, CD146, CD73, and CD29 and the mesodermal marker vimentin (VIM), as well as the embryonic stem cell markers NANOG, OCT4, Musashi, and Nestin and embryonic renal marker Pax2, but lacked differentiative epithelial markers such as Cytokeratin. These cells were clonogenic and could generate spheres in specific cell culture medium, which is considered an important characteristic of CSCs. They could differentiate* in vitro* and* in vivo* into epithelial cells. CD105+ cells displayed tumor-initiating activity, and as few as 100 cells could generate serially transplantable carcinomas in immunodeficient mice containing few CD105+ tumorigenic cells and a large number of differentiated CD105− cells. They did not express CD133, a known marker of adult human tubular progenitor cells [28, 29]. The same authors also found that extracellular vesicles (EV) released by renal CD105+ CSCs, but not EV derived from a more differentiated CD105− tumor cell population, were able to modify tumor microenvironment and to promote development of a lung premetastatic niche [30]. Recently, they further characterized EV and observed that they favor tumor progression and metastases and are able to modulate the behavior of monocyte-derived dendritic cells and impair T-cell immune response by a mechanism involving HLA-G to the same extent as CSCs [31].

Through automated digital assessment of intratumoral microvascular density, Dubinski et al. showed that CD105 is an unfavorable prognostic marker in ccRCC [32]. Indeed, patients with higher CD105 expression level had significantly shorter progression-free survival as well as a higher tumor stage. CD105 is an important indicator of clinical outcome and could become a valuable therapeutic target following further investigation.

#### 2.1.2. CD133

CD133 is a five-transmembrane domain glycoprotein, and its true function still remains elusive [33]. Human CD133 was first isolated from hematopoietic stem cells by a specific monoclonal antibody that recognized a specific epitope (clone AC133). CD133 currently serves as a useful marker for the isolation of many different types of stem and progenitor cells in adult human tissues, even for clinical purposes [33]. However, only antibodies recognizing the epitopes localized in the second extracellular loop, such as the AC133 and 293C3 clones used to identify human renal progenitors, are suitable for recognition of stem cells and progenitor cells, while other antibodies do not specifically recognize stem cells but also differentiated epithelial cell types [33]. As previously mentioned, CD133 is also a marker of renal progenitor cells in adult human kidney, and resident RCC CD133+ cells have been shown to promote tumor vascularization and angiogenesis [34]. Bruno et al. sorted CD133+ and CD133− cells from RCC patients and showed that CD133+ cells alone did not induce tumor formation in immunodeficient mice, but cotransplanted with cells from the K1 RCC cell line they led to enhanced development and growth of tumors. This effect was not imputed to the tumorigenic nature of CD133+ cells as identical effects were observed using CD133+ cells from normal kidney, and it was speculated that the CD133+ cells present in the tumor could have migrated from healthy kidney tissue. Interestingly, the neovasculature was from human origin, as shown by the presence of human HLA class I and CD31, indicating CD133+ cells as their origin, results corroborated by fluorescence* in situ* hybridization for expression of human chromosome X [34]. Galleggiante et al. identified and characterized a population of ccRCC-derived CD133+/CD24+ CSCs [35]. Gene expression profile identified copper transport protein 2 (CTR2) as a membrane marker for this neoplastic population. CTR2 in RCC patients had an important role in cisplatin-based resistance. CD133+/CD24+/CTR2+ cells did not express mesenchymal markers and were more undifferentiated than tubular adult renal progenitor cells. These cells presented* in vitro* self-maintenance and differentiating capabilities and could induce an angiogenic response* in vivo*. Lindgren et al. isolated a population of ALDH^high^ cells from adult human renal cortical tissue, which also expressed CD133 and VIM [19, 29]. They showed that this CD133+/VIM+ population of tubular-committed progenitors demonstrate a significant transcriptional similarity with pRCC and coexpress VIM, keratin 7 (KRT7), and KRT19, a pattern characteristic of pRCC. Additionally, VIM+/KRT7+/KRT19+ cells were observed in cortical adenomata, benign renal neoplasms. These results suggest that pRCC develops from these progenitor cells and that cortical adenomas may represent a benign intermediate step during this oncogenic process.

Clinical significance of CD133 expression in human RCC is inconsistent and varies greatly between studies [24]. da Costa et al. used an anti-CD133 polyclonal antibody, which stained diffusely differentiated epithelial structures in embryonic as well as adult kidneys, and found that patients in the CD133 low-expression group had a higher probability of death from RCC and disease progression [36]. Conversely, D'Alterio et al. used an anti-CD133/1 monoclonal antibody that selectively recognized renal progenitors and did not see any correlation with the clinical pathological features or patient prognosis [37]. However, another two studies which used the anti-CD133 monoclonal antibody showed contrasting results. Zhang et al. observed that CD133 expression correlated with tumor grade, stage, histological type, and tumor location [38]. Prognosis of patients with CD133^high^, CD44^high^, positive vasculogenic mimicry, and low microvessel density was worse than that of patients with CD133^low^, CD44^low^, negative vasculogenic mimicry, and high microvessel density. Kim et al. reported that high levels of CD133 expression were observed in ccRCC with more differentiated morphology and were associated with a macro-/microcystic pattern, nonsarcomatoid changes, and nonmetastatic disease and therefore would consider it as a favorable prognostic marker [39]. A follow-up study by the same group showed that CD133 may serve as a favorable prognostic marker in pRCC, as it correlated with small tumor size, low Fuhrman nuclear grade, and prolonged disease-specific survival [40]. Further studies on the significance of CD133 expression in RCC are thus necessary.

#### 2.1.3. CXCR4

CXCR4 is an alpha-chemokine receptor specific for stromal-derived factor-1 (SDF-1, also called CXCL12), a molecule endowed with potent chemotactic activity. CXCR4 has been found to be a prognostic marker in various types of cancer and plays a role in the cell proliferation and migration of cancer cells [41]. The CXCL12/CXCR4 axis forms critical communication bridges between tumor cells and stromal cells to create a permissive microenvironment for tumor growth and metastasis. Schrader et al. analyzed CXCL12*α*/CXCR4 expression and function in human RCC cell lines (A-498, Caki-1, Caki-2, and HA-7), patient RCC samples, and corresponding normal kidney tissue [42]. They observed that none of the four RCC cell lines expressed CXCL12, while A-498 cells expressed CXCR4. More importantly, RCC samples showed a decreased expression of CXCL12 and increased expression of CXCR4, compared to their respective adjacent normal kidney tissue, revealing a role in tumor progression. Gassenmaier et al. compared two RCC cell lines, RCC-26 and RCC-53, in their capacity to form spheres* in vitro* and to establish tumors* in vivo* [43]. They observed differences in levels of chemokine expression, as CXCR4 was present only in the more tumorigenic cell line RCC-53. CXCR4+ cells presented CSC characteristics, such as increased resistance to tyrosine kinase inhibitors, higher sphere-forming ability, and tumor growth-inducing potential* in vivo,* and expressed high levels of stem cell-associated makers NANOG, OCT3/4, and SOX2. Downregulation of CXCR4 expression by small interfering RNA or pharmacological inhibition by AMD3100 hindered sphere formation and reduced the viability of CXCR4+ cells [43]. Recently, Micucci et al. showed that the enhanced self-renewal activity of the CXCR4+ spheres was preceded by the upregulation of hypoxia-inducible factor 2*α* (HIF2*α*) in the RCC cell lines Caki-1, Caki-2, 786-O, and 769-P [44]. Knockdown of HIF2*α* abrogated CXCR4 expression and sphere formation, while inhibition of HIF2*α* abolished tumor growth* in vivo*, revealing the crucial role of HIF2*α* activation in CSC expansion.

Numerous studies show that an elevated CXCR4 expression in tumor samples from RCC patients is correlated with poor outcome [37, 45–47]. Additionally, CXCR4 is significantly related to the biological features of the tumor stage, including stage, Fuhrman grade, and clinical presentation [48].

#### 2.1.4. CD44

The CD44 antigen is a cell surface glycoprotein involved in cell-cell interactions and cell adhesion and migration. A major receptor for hyaluronic acid, CD44, is an extensively described CSC marker in several human carcinomas [49]. In the human embryonic cell line 293T, Debeb et al. described CD44+/CD24− cells with several CSC features [50]. Although the nature of CSCs is still the subject of debate, CD44+ human carcinomas are highly malignant and resistant to therapy, and the presence of CD44 has been shown to confer increased metastatic potential, properties that are frequently associated with CSCs [38, 49]. CD44 is closely associated with proliferation, metastasis, cancer recurrence, and prognosis in RCC [51]. Lim et al. suggested that CD44 expression in RCC provides useful prognostic information both in primary and in metastatic RCC and may help in determining the appropriate therapy [51]. A meta-analysis of the literature performed by Li et al. revealed that elevated CD44 expression is a poor prognostic marker for five-year overall survival and correlates with high Fuhrman grade and recurrence [52]. A possible mechanism of upregulation of CD44 expression in ccRCC has been proposed in 2016 by Ma et al. [53]. They observed that about a third of tumor samples expressed CD44, with no correlation with clinical outcome, but associated with a high density of tumor-associated macrophages.* In vitro* experiments using RCC cell lines and human macrophages demonstrated that CD44 expression increased following direct coculture with macrophages. Silencing or suppression by NF-*κ*B inhibitors of TNF-*α* on macrophages abolished the increased CD44 expression in RCC. This study suggests that TNF-*α* derived from tumor-associated macrophages is linked to CD44 upregulation via NF-*κ*B signaling in ccRCC [53]. Even though the nature of CD44 as CSC marker is unclear, its role in RCC tumorigenicity remains noteworthy and further investigation might reveal interesting clinical applications.

### 2.2. Functional Assays

Identifying selective CSCs could be a difficult task, and several functional assays have been set up to circumvent this obstacle.

#### 2.2.1. Sphere-Forming Capacity

Zhong et al. proposed to select CSCs from the SK-RC-42 RCC cell line on their capacity to form spheres in a serum-free medium, in the presence of epithelial growth factor and basic fibroblast growth factor [54]. This cell population expressed stem cell markers (OCT4, NANOG, BMI, and *β*-catenin) and was more resistant to chemotherapeutic agents and radiation than monolayer adherent cells. Sphere-forming cells displayed a distinct immunophenotype, as they did not express MHC-II, CD80, FAS, and natural killer (NK) activating receptors, indicating that they could contribute to T-cell and NK cell immune suppression. Surprisingly, both sphere-forming and monolayer adherent cells expressed comparable levels of CD133, CD44, and CD24 markers, while CD105 levels were higher in monolayer adherent cells [54]. Using the same approach, Lichner et al. showed that RCC spheres exhibit CSC properties, including self-renewal, high tumorigenicity, and differentiation capacity, as well as the expression of OCT4, NANOG, KLF4, and LIN28 [55]. Interestingly, inhibition of miR-17 enhanced RCC sphere formation, while overexpression of miR-17 hampered sphere formation, through modulation of the TGF-*β*-EMT axis [55, 56]. miR-17 belongs to an oncogenic microRNA (miRNA) cluster which is essential for development and homeostasis [57]. However, the dual oncogenic/tumor suppressor function of miR-17 and the other miRNAs of the family has been suggested in various tumor types, including RCC, and will require close scrutiny [58, 59]. miRNAs have an important role in posttranscriptional gene regulation and have been shown to be required for the maintenance of normal pluripotent embryonic stem cells in mice [60]. They have been described in several other types of solid tumors, such as breast cancer, and further investigation of their role in RCC tumor initiation, therapy resistance, progression, relapse, and metastasis will certainly help understand tumor biology [61].

#### 2.2.2. Side Population Sorting

Addla et al. isolated CSCs by sorting the side population (SP) of cells that take up Hoechst 33342 dye, by flow cytometry analysis [62, 63]. This technique was previously described to identify hematopoietic stem cells (HSCs). HSCs can be distinguished by their ability to form a SP, as they are able to quickly carry out dyes efflux owing to the presence of specific membrane transporters on their surface (such as ABC transporters), a property typically associated with stemness [64]. About 6% of RCC cells could be defined as SP, and these cells expressed CD105 but not CD133 and were able to proliferate and differentiate, as well as grow into spheroids. However, the cells were found to be both in G0 and in G1 phase of the cell cycle, suggesting heterogeneity within the SP. Relationship between SP and CSCs has been questioned in several types of neoplasms, such as in glioblastoma, where SP does not contribute to self-renewal and tumorigenicity attributed to CSCs [65, 66]. Huang et al. applied this method to identify SP in five human RCC cell lines. Flow cytometry analysis revealed that the 769P cell line contained the largest SP, which presented CSC characteristics such as the ability to proliferate, self-renew, and differentiate, as well as strong resistance to chemotherapy and radiotherapy that could be linked to the ABCB1 transporter.* In vivo* serial tumor transplantation in immunodeficient mice showed that 769P SP cells formed tumors [67]. Hughes et al. suggested combining Hoechst SP detection with synchrotron radiation-Fourier transform infrared spectroscopy in order to measure discrete differences in the biochemistry of small numbers of single cells [68]. They showed that SP cells were very small, consisting of a nucleus and limited cytoplasm, relative to the remaining renal cells. A similar SP identification approach consists in evaluating the cell's ability to carry out toxins efflux using Rhodamine 123 (Rho123). Lu et al. separated Rho123^high^ from Rho123^low^ in the 786-O RCC cell line and showed that the Rho123^high^ population formed a small subset of cells with higher proliferative activity, long-term differentiation potential, resistance to radiation, increased colony-forming capacity, and high tumorigenicity compared to Rho123^low^ cells [69]. Rho123^high^ could therefore be considered to be CSCs, even though they lacked CD105 expression.

Recently, SP cells from the RenCa RCC cell line were genetically modified to knock out (KO) DnaJ (Hsp40) homolog, subfamily B, member 8 (Dnajb8) and investigate its role in the tumorigenicity of RCC [70]. The authors confirmed a previously described role for this heat shock protein in the maintenance of RCC CSCs, as Dnajb8 KO cells showed reduced ratios of SP cells and reduced sphere-forming capacity [70, 71].* In vivo* single-cell transplantation assay revealed a role for DNAJB8 in tumor initiation, while* in vitro* experiments did not indicate a function in stress responses [70]. SP cells could be used as a potent tool to assess gene functions in a wide range of experiments. Additionally, DNAJB8 emerged as a new potential CSC marker. However, no clinical significance data is available so far for either SP cells or DNAJB8.

#### 2.2.3. Aldehyde Dehydrogenase 1 Enzymatic Activity

Recent evidence suggests that enhanced aldehyde dehydrogenase (ALDH) activity is a hallmark of CSCs, assessable by the ALDEFLUOR assay [72]. Debeb et al. cultured and passaged HEK 293T cells as spheres in serum-free stem cell-promoting medium [50]. They observed a larger number of ALDH+ cells in spheres compared to cells growing in monolayer. As mentioned earlier, these cells were also CD44+/CD24− and exhibited a CSC-like phenotype, that is, resistance to radiation and expression of higher levels of stem cell survival signaling including *β*-catenin, Notch1, and Survivin. Moreover, spheres have increased expression of mesenchymal genes including VIM, N-cadherin, Zeb1, Snail, and Slug as well as prometastatic genes RhoC, Tenascin C, and MTA1. Additionally, levels of miRNAs associated with self-renewal and metastasis formation were significantly decreased in the spheres. 293T cells cultured as spheres represent an important research tool for studying the molecular and biological mechanisms of CSCs and for testing and developing new targets for cancer therapy [50]. In ACHN and Caki-2 RCC cell lines, ALDH^high^ cells displayed several CSC properties* in vitro*, that is, clonogenic and self-renewal ability and increased expression of OCT3/4A, NANOG, and Pax2. ALDH^high^ cells had higher tumorigenicity* in vivo* [73]. Ueda et al. studied the ALDH enzymatic activity of SP from ACHN and KRC/Y RCC cell lines [74]. In the metastatic ACHN cell lines, they observed that ALDH+ cells formed about 15% of the total number of cells and had higher sphere-forming capacity, self-renewal ability, and tumorigenicity than ALDH− cells. Furthermore, SP cells were enriched in ALDH+ cells compared to non-SP cells, suggesting a certain correlation between ALDH enzymatic activity and SP. However, in the primary KRC/Y cell line, only 6.5% of the cells were ALDH+ and this proportion was similar in SP and non-SP. The population of CD133+/VIM+ cells described by Lindgren et al. and mentioned earlier were isolated from ALDH^high^ adult human renal cortical tissue, underlining the importance of combining several isolation techniques [29].

While Ozbek et al. reported that ALDH1 expression was correlated with tumor grade but not with tumor stage in patients with RCC, Abourbih et al. observed that ALDH1 expression did not vary significantly based on tumor stage or grade and did not correlate with progression-free survival [75, 76]. Further investigations are necessary to determine the relationship between RCC clinical prognosis and ALDH1.

## 3. Cancer Stem Cell-Targeted Therapies

As previously stated, tumors from RCC patients show resistance to chemotherapy and radiation and are weakly responsive to immunotherapeutic agents such as interferon *α* and interleukin-12. While antiangiogenic drugs like tyrosine kinase inhibitors (TKI) have significantly improved outcome in patients with metastatic disease, the majority still presents resistance over time as the tumor develops evasion mechanisms in response to vascular endothelial growth factor (VEGF) inhibition [77]. Varna et al. have demonstrated the involvement of CD133+/CXCR4− CSCs in that process [78]. In perinecrotic areas where CSCs were numerous and predominant, the VEGF inhibitor sunitinib was able to generate resistance to its own therapeutic effect via induced hypoxia, which promotes tumor aggressiveness [18]. Indeed, hypoxia leads to the activation of HIF, which causes adaptive changes within cancer cells and aggressive behavior contributing to tumor progression and resistance and conducting to poor prognosis [44].

**Figure 3:**
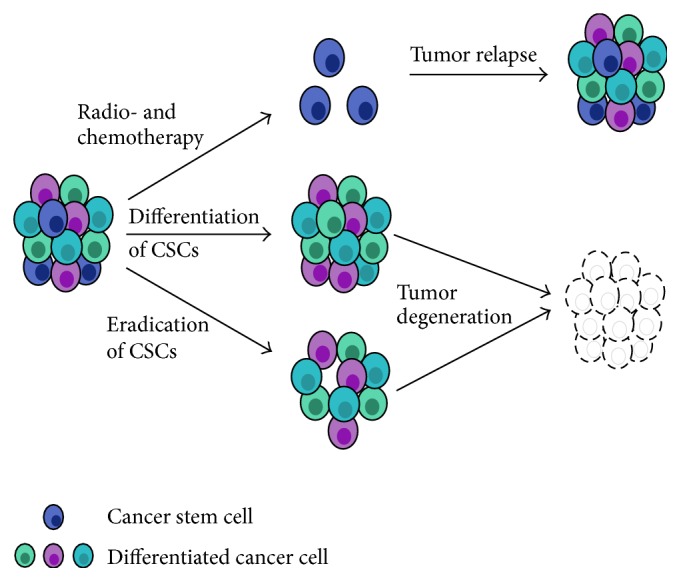
Therapies to target radio- and chemoresistant cancer stem cells. The presence of cancer stem cells (CSCs) in the tumor can lead to a relapse following conventional therapy. Newly developed strategies propose to either differentiate or eradicate CSCs, leading to the degeneration of the tumor.

Therefore, it might be of great interest to develop new therapeutic drugs or immunotoxins that target CSCs, based on molecular mechanisms that regulate stem cell properties (Figure 3) [16].

### 3.1. Interleukin-15

The identification of reliable inducers of CSC differentiation could facilitate the elaboration of efficient strategies for eradicating CSCs. Azzi et al. demonstrated that interleukin-15 (IL-15), a regulator of kidney homeostasis, could induce the differentiation of CD105+ CSCs from human RCC [23, 79]. Previously published data on IL-15 and IL-15R*α* KO mice showed that IL-15 was an autocrine survival factor for renal tubular epithelial cells [80, 81]. CD105+ CSCs treated with IL-15 lost the expression of stem cell markers and tumor-initiating and sphere-forming ability. On the other hand, they gained epithelial markers as well as functional epithelial properties such as polarity and transmembrane resistance [79]. Most importantly, CD105+ CSCs became sensitive to the chemotherapeutic drugs vinblastine and paclitaxel. By contrast, RCC neither secrete the cytokine nor express—both* in vivo* and* in vitro*—the IL-15R*γ* chain and JAK3 [82]. Another* in vitro* study showed that IL-15 upregulated E-cadherin expression through *ϒ*c chain signaling pathway on renal epithelial tubular cells and blocked their EMT [83].

A recently completed phase I study recorded on the National Institutes of Health (NIH) registry (NCT01021059 on https://clinicaltrials.gov/) proposed to administer intravenous recombinant human IL-15 in adults with refractory metastatic malignant melanoma and metastatic renal cell cancer. Preliminary results showed the safety and feasibility of the study [84]. IL-15 administration markedly altered the homeostasis of lymphocyte subsets in blood, in particular NK cells and *γδ* cells and CD8 memory T cells. To reduce toxicity and increase effectiveness of the treatment, dosing strategies have been modified, including continuous intravenous infusions and subcutaneous IL-15 administration.

### 3.2. mTOR Inhibitors

The mammalian target of rapamycin (mTOR) is a serine/threonine protein kinase, member of the large phosphatidylinositol 3-kinase- (PI3K-) related kinase family. It is known to regulate cell growth and cell proliferation in stem cells [85]. mTOR pathway activation leads to constitutive HIF-1*α* expression, an important signaling in the pathogenesis of RCC, as well as the expression of cell-cycle regulators c-myc and cyclin D1 [86–89]. mTOR inhibitors have been shown to inhibit both tumor cell proliferation and angiogenesis [90, 91]. Numerous recent studies have demonstrated the links between the PI3K/Akt/mTOR signaling pathway and CSC biology [92]. Interestingly, experiments on neuroblastoma showed that rapamycin targets specifically CSCs [93]. Similar data confirmed that mTOR inhibitors eradicated CSCs in nasopharyngeal carcinoma, colon cancer, and pancreatic cancer [94–96]. However, contrasting studies underline the putative role of CSCs in mTOR inhibitor-mediated resistance to RCC treatment, in particular in ccRCC [86]. A recent study detailed this resistance mechanism in breast cancer [97]. Accumulative evidence shows that mTOR is an important pathway in carcinogenesis, and a better understanding of the effects of mTOR inhibition in RCC will lead to more successful therapies.

On the NIH database, as many as 36 phase I, II, and III clinical trials, ongoing or completed, propose to use mTOR inhibitors to treat RCC. The phase III trial on treatment-resistant patients with metastatic RCC showed safety and benefits of everolimus over placebo (NCT00410124). Multiple combination treatments have been proposed to lower everolimus toxicity while maintaining a sufficient efficiency [91]. The advantage of such an approach is that it can modulate different signaling pathways involved in cancer biology and target both CSCs and cancer cells for a more global effect.

### 3.3. Bone Morphogenetic Protein 2

The bone morphogenetic protein 2 (Bmp-2) encodes a member of the TGF-*β* superfamily. It plays an important role in the development of bone and cartilage, as well as in the regulation of various cellular processes including cell differentiation, proliferation, morphogenesis, cellular survival, and apoptosis [98]. BMP-2 has been reported to either stimulate or inhibit tumor growth, depending on cancer type [99]. Wang et al. showed that BMP-2 could inhibit tumorigenicity of CSCs in human osteosarcoma OS99-1 cells, inhibit tumor growth of human RCC, and induce bone formation [100–102]. In a follow-up study, the authors evaluated whether BMP-2 can be used to block the tumor-initiating ability of human renal ALDH+ CSCs* in vitro* and induce bone formation* in vivo* [99]. They found that BMP-2 inhibited CSC growth, downregulated the expression of stem cell markers, and upregulated the transcription of osteogenic markers. ACHN and Caki-2 RCC cells implanted in immunodeficient mice developed into large tumors, while animals treated with BMP-2 presented limited growth and important bone formation, indicating BMP-2 as a potential new drug targeting CSCs in RCC. A recent study by Mitsui et al. revealed that impaired regulation of Bmp-2 via epigenetic pathways was associated with RCC pathogenesis and confirms the usefulness of BMP-2 as a molecular marker for designing improved diagnostic and therapeutic strategies for RCC [103]. Several clinical trials involving BMP-2 treatment have been registered, primarily for patients with fractured and degenerative disk disease, but none for cancer patients.

### 3.4. Other Possible Therapeutic Targets

Numerous other strategies have been proposed to target CSCs but have not been experimented with on RCC so far [25, 104, 105]. They include targeting ATP-driven efflux transporters or cell surface markers, inhibiting CSC-related signaling pathways, inhibiting autophagy signaling in CSCs, increasing bioavailability of CSC-specific agents, delivering CSC-specific chemotherapeutics or nucleic acid drugs, and using CSC-targeted nanocarriers. Among those, a few stand out and deserve attention.

#### 3.4.1. ATP-Driven Efflux Transporter Inhibition

As previously stated, CSCs present high levels of ABC transporters, which might be involved in drug resistance by decreasing the cellular accumulation of therapeutic agents [106]. Several groups have attempted to eradicate CSCs using the low molecular weight inhibitors fumitremorgin C and tryprostatin or monoclonal antibodies such as cyclosporin A, VX710, or tariquidar [104, 107, 108]. Their use in clinical settings has been hindered by low inhibition efficiency and elevated toxicity to healthy cells.

#### 3.4.2. Cell Surface Marker Inhibition

Monoclonal antibodies and inhibitors have been proposed to block CSC surface markers. Jin et al. used an activating antibody directed to CD44 to inject into immunodeficient mice transplanted with acute myeloid leukemia and observed lower leukemic repopulation [104, 109]. Therapies against CD133 have been successfully used to treat lung cancer, glioblastoma, and liver cancer [110–112]. Due to the few experiments performed using this strategy, further studies must be carried out to validate this approach.

#### 3.4.3. Salinomycin Treatment

Salinomycin is a biologically active substance isolated from the culture broth of a strain of* Streptomyces albus*. Gupta et al. identified salinomycin as a selective inhibitor of human breast CSCs by high-throughput screening [113, 114]. Successive studies demonstrated that salinomycin could kill CSCs in different types of human cancers including gastric cancer, lung adenocarcinoma, osteosarcoma, colorectal cancer, squamous cell carcinoma, and prostate cancer, most likely by interfering with ABC drug transporters, the Wnt/*β*-catenin signaling pathway, and other CSC pathways. Promising results from preclinical trials in human RCC show that salinomycin is able to effectively eliminate CSCs and to induce partial clinical regression of heavily pretreated and therapy-resistant cancers [113, 115].

#### 3.4.4. Nanomedicine-Based Therapies

Recently, nanomedicine-based therapies have been developed to target CSCs [104]. Nanoparticles can be used as high-capacity carriers for chemotherapeutic or nucleic acid drugs and can accumulate at tumor sites through two different mechanisms: a passive one, that is, enhanced permeation retention due to the higher porosity of the vasculature and impaired lymphatic drainage, or an active one, due to the presence on the nanoparticles of molecules of high affinity for the receptors exclusively overexpressed on the surface of CSCs [116, 117]. As an example, it has been proposed to incorporate salinomycin into nanocarriers in order to lower its toxicity. Yao et al. developed a drug delivery system which targets specifically gastric CSCs [118]. Indeed, chitosan-coated single wall carbon nanotubes loaded with salinomycin functionalized with hyaluronic acid could selectively eliminate CD44+ gastric CSCs* in vitro*. As a combination therapy, Zhang et al. used octreotide-modified paclitaxel-loaded PEG-b-PCL polymeric micelles and salinomycin-loaded PEG-b-PCL polymeric micelles to eradicate breast cancer cells [119]. While paclitaxel eliminated the bulk of cancer cells, salinomycin targeted CSCs, resulting in a stronger antitumoral activity than either drug separately* in vitro* and* in vivo*. Numerous studies in a wide range of cancers showed the efficiency of nanoparticle-based therapies to target CSCs [104].

Nanoparticles are becoming a prominent player in the fight against tumors, as more than 150 clinical trials registered with the NIH propose this innovative technology combined with more conventional therapies to treat cancer.

Despite important progress being made in developing CSC-targeted therapies, very few discoveries have been translated to a clinical setting, underlining the complexity of the mechanisms involved in carcinogenesis. However, the major mobilization of the scientific community suggests that this approach is worth exploring notwithstanding the numerous setbacks.

## 4. Conclusions

A vast body of evidence supports the existence within renal tumors of CSCs with self-renewal and multidifferentiation properties. Various approaches have been described to successfully isolate and characterize CSCs, leading to the identification of a variety of CSCs and suggesting that several subpopulations of CSCs may coexist within a heterogeneous tumor. Combination of stem cell markers with functional assays permits a better identification of a smaller population enriched in CSCs, but important limitations have been observed [22, 120]. For example, functional assays present important variations in their staining protocols, from dye concentration to incubation time, leading to important discrepancies in the results [66]. Additionally, culture conditions as well as cell detachment methods in* in vitro* experiments must be optimized and standardized in order not to affect CSC phenotype [121–123]. Despite the aforementioned caveats, identification of CSCs led to a better understanding of carcinogenesis.

It is worth noticing that the techniques developed to isolate and study CSCs are all performed* in vitro*, eventually followed by implantation of the cells into immunodeficient mice to test their tumor forming capacity. However, most studies involving RCC patient samples failed to verify that CSCs could be xenografted into immunodeficient mice and could produce serially transplantable tumors that recapitulate the original tumoral tissue. Interestingly, Hasmim et al. proposed an alternative strategy for identifying renal CSCs by adapting, to* in vitro* culture, primary cell suspensions from serial patient-derived xenografts obtained by implanting RCC samples in immunodeficient mice [124]. They isolated 3 different CSC subsets of cells, which presented different characteristics and confirmed the heterogeneity of the CSC population. Indeed, all 3 populations were ALDH+ and formed serial spheroids but expressed different combinations of stem cell markers (CD133, CD105, CD146, CD29, OCT4, NANOG, and Nestin) and the non-CSC tumor marker E-cadherin. Admirably, all 3 subsets developed serial tumors in SCID mice and therefore successfully encompassed all characteristics of CSCs. This approach is gaining attention within the scientific community, as the use of patient-derived xenografts will allow in the future the development of personalized medicine, that is, unique treatment regimens for individual patients [125].

While their clinical significance remains elusive at the moment, CSCs undoubtedly play an important role in tumor resistance to radiotherapy and chemotherapy. To circumvent this drawback in conventional therapy, several research groups are taking advantage of CSCs unique properties to design new drug compounds that selectively target CSCs. A few therapies, such as IL-15 and mTOR administration, are already being tested in clinical trials to treat RCC, providing new prospects for patients.
